# Preliminary Real-World Evidence Supporting the Efficacy of a Remote Neurofeedback System in Improving Mental Health: Retrospective Single-Group Pretest-Posttest Study

**DOI:** 10.2196/35636

**Published:** 2022-07-08

**Authors:** Jocelyne C Whitehead, Ron Neeman, Glen M Doniger

**Affiliations:** 1 Integrated Program in Neuroscience McGill University Montreal, QC Canada; 2 Myndlift Ltd Tel Aviv Israel

**Keywords:** EEG biofeedback, remote care, neurofeedback, attention-deficit/hyperactivity disorder, delta/alpha ratio

## Abstract

**Background:**

Neurofeedback training (NFT) has been shown to be effective in treating several disorders (eg, attention-deficit/hyperactivity disorder [ADHD], anxiety, and depression); however, little is currently known regarding the effectiveness of remote NFT systems.

**Objective:**

This retrospective study provides real-world data (N=593) to assess the efficacy of app-based remote NFT in improving brain health and cognitive performance.

**Methods:**

Improvement was measured from pre- to postintervention of in-app assessments that included validated symptom questionnaires (the 12-item General Health Questionnaire, the ADHD Rating Scale IV, the Adult ADHD Self-Report Scale, the 7-item Generalized Anxiety Disorder scale, and the 9-item Patient Health Questionnaire), a cognitive test of attention and executive functioning (ie, continuous performance task), and resting electroencephalography (EEG) markers. Clinically significant improvement was evaluated using standard approaches.

**Results:**

The greatest improvement was reported for the anxiety questionnaire, for which 69% (68/99) of participants moved from abnormal to healthy score ranges. Overall, adult and child participants who engaged in neurofeedback to improve attention and executive functions demonstrated improved ADHD scores and enhanced performance on a cognitive (ie, response inhibition) task. Adults with ADHD additionally demonstrated elevated delta/alpha and theta/alpha ratios at baseline and a reduction in the delta/alpha ratio indicator following neurofeedback.

**Conclusions:**

Preliminary findings suggest the efficacy of app-based remote neurofeedback in improving mental health, given the reduced symptom severity from pre- to postassessment for general psychological health, ADHD, anxiety, and depression, as well as adjusted resting EEG neural markers for individuals with symptoms of ADHD. Collectively, this supports the utility of the in-app assessment in monitoring behavioral and neural indices of mental health.

## Introduction

### Background

Neurofeedback training (NFT) is considered a primary or supplementary treatment for a number of disorders, including attention-deficit/hyperactivity disorder (ADHD) [1-5], anxiety [6-9], and depression [7,8,10]. The American Academy of Pediatrics [11] provided a “level 1 best support” rating of NFT as a safe and effective evidence-based therapy for childhood ADHD. Nonetheless, several significant barriers prevent patients from receiving quality neurofeedback therapeutics; for example, electroencephalography (EEG) systems are expensive, complex, and often only accessible at health care clinics. A recent pilot study [12] provided encouraging evidence for the efficacy of therapist-guided NFT, suitable for remote home-based use. Findings showed improved ADHD symptomatology in a small cohort of children after 9 weeks of NFT. The system was designed as an affordable convenient wireless alternative to clinic-based EEG. NFT users regulate neural activity through operant conditioning, which can lead to morphological changes in the brain [13,14] and calmer, more focused cognitive, affective, and physical functioning. Currently, little is known about the effectiveness of NFT systems in the field [15]; therefore, this retrospective open-label pilot study offers real-world data supporting the efficacy of remote NFT in improving brain health.

### Mental Health Improvement in Real-World Settings

**Figure 1:**
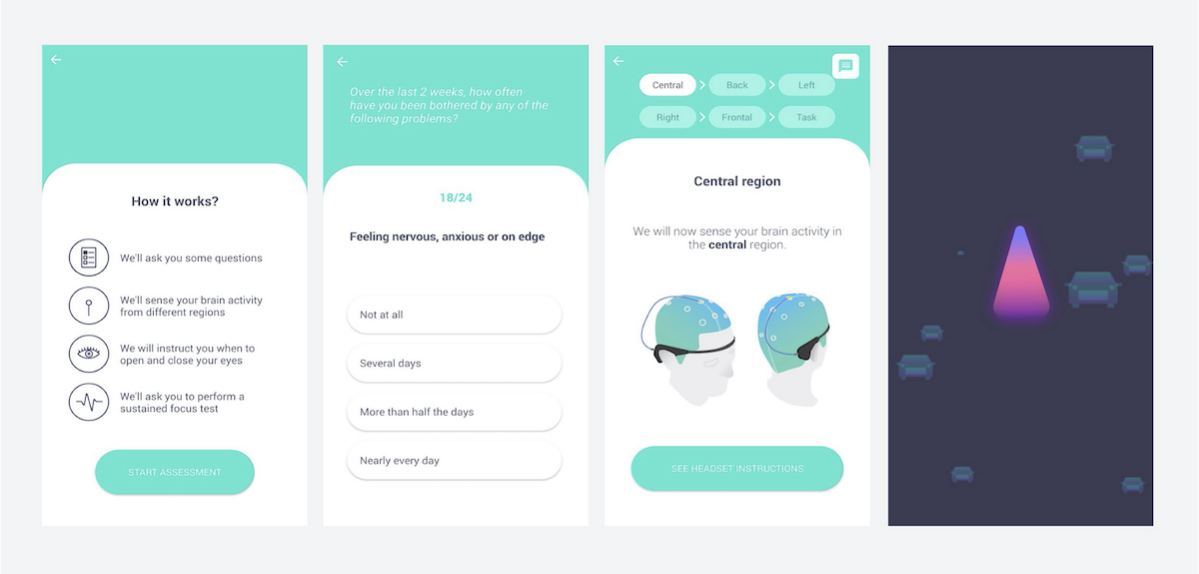
Myndlift in-app assessment screens. From left to right: introduction, symptom questionnaires, resting electroencephalography assessment, and cognitive task.

Unlike standard EEG systems, Myndlift is an easy-to-use tool for patients and clinicians (Figure 1). While wearing the validated EEG headband (Muse; InteraXon [16,17]) containing four dry recording electrodes (ie, anterior frontal [AF] 7, AF8, temporal pole [TP] 9, and TP10), one ground electrode, and one auxiliary wet electrode, the patient trains with an Android or iOS app linked to the headset by Bluetooth, which delivers visual and auditory feedback during YouTube videos or specialized games. When patients’ brain waves are in the desired range, positive feedback is delivered. A therapist can set or adjust the training protocol and monitor progress remotely via a cloud-based web service. The device incorporates an app-based assessment, lasting approximately 40 minutes, completed prior to NFT (ie, baseline) and periodically over the intervention period for longitudinal tracking of improvement.

Real-world studies provide external validity and accurately represent the heterogeneity of a patient population [18]. From the app, real-world data were collected from more than 500 participants on outcome measures, including pre- and postintervention assessments of validated symptom questionnaires, a cognitive test of attention and executive functioning (ie, continuous performance task [CPT]), and resting EEG markers. An efficacious system could serve as a reliable, cost-effective solution for users. In-clinic NFT costs approximately US $150 to $200 per session, with a minimum of 30 to 40 sessions typically recommended. In contrast to a cost-per-session model, remote NFT could offer monthly charges, ranging from US $200 to $500.

### EEG Neuromarkers of ADHD

Given the success of neurofeedback for child ADHD, more adults with ADHD are turning to NFT for treatment. Currently, 6.76% of adults worldwide—translating to 366.3 million people—are affected [19]. ADHD is commonly recognized as a hypoaroused brain state [20]. In recent years, EEG measures have provided supporting evidence for popular theoretical models of hypoactivation [21] related to core symptoms of hyperactivity, inattention, and impulsivity [22]. The hypoarousal state is best localized to frontal and posterior regions [23] (ie, neuroanatomical structures subserving attentional networks [24,25]). EEG patterns of ADHD in children are characterized by elevated low-frequency power (ie, primarily theta) and reduced *relative* high-frequency power (ie, alpha and beta) [23,26-28], or an elevated ratio of the two (ie, low to high frequency). The theta/beta ratio (TBR) is the most common form of NFT in treating ADHD [29,30]; however, inconsistencies in the literature suggest that TBR [31,32] may not be reliable as a diagnostic measure [33]. This may reflect EEG heterogeneity across ADHD-diagnosed individuals (eg, Diagnostic and Statistical Manual of Mental Disorders, Fifth Edition [DSM-5] subtypes; psychiatric comorbidities; age; and sex) [34,35]. For instance, although theta and beta power differences are evident in child ADHD [31,36], a recent review [37] suggested that theta and alpha frequencies may be more reliable markers for adults. Notably, most adult studies emphasize group differences in alpha power during eyes-closed conditions [38-43], while more recent work has identified elevated theta and delta power in adults with ADHD [44,45]. Given this evidence, this study investigates whether TBR versus the delta/alpha ratio (DAR) or the theta/alpha ratio (TAR) are biomarkers for adult ADHD. Overall, the study evaluates evidence for improvement in mental health via symptom questionnaires, a CPT, and hypothesized EEG markers. Findings have implications for the benefits of NFT and efficacy of a remote home-based system.

## Methods

### Participants

Participants, 13 years of age or older, signed up through their clinician or a clinician suggested by Myndlift and completed NFT at home or in clinic in a clinical care context. Informed consent was provided through the app, allowing participants’ anonymized data to be used for research. Data were included for analysis if baseline (ie, preintervention) assessment was conducted after 7 or fewer NFT sessions (ie, attributed to in-app NFT tutorial). For analyses of improvement, postintervention sessions occurred 30 to 180 days after baseline with 20 or more NFT sessions completed [46]. An average of 1 or more NFT sessions per week was required for inclusion, given that effective neurofeedback requires consistency [47,48], irrespective of the neurofeedback protocol used. Data were collected via the app.

### Ethical Considerations

Procedures were reviewed by an independent Institutional Review Board (IRB)—Pearl IRB—who permitted IRB exemption for analyses of data previously collected and deidentified, following the guidelines of the Declaration of Helsinki.

### Neurofeedback Protocol

Participants performed neurofeedback protocols (Multimedia Appendix 1) that were customized by their clinicians and consistent with current literature [49].

### Procedure and Outcome Measures

#### Symptom Questionnaires

The in-app assessment includes 14 brief standardized questionnaires commonly used to screen for mental health conditions. In this study, data were reported for the following five questionnaires completed at baseline and follow-up by at least 25 participants: the 12-item General Health Questionnaire (GHQ-12) [50], the ADHD Rating Scale IV (ADHD-RS-IV) [51], the Adult ADHD Self-Report Scale (ASRS) for DSM-5 [52], the 7-item Generalized Anxiety Disorder scale (GAD-7) [53], and the 9-item Patient Health Questionnaire (PHQ-9) [54]. For each questionnaire, participants filled out self-report measures based on frequency of symptom occurrence using a 4- or 5-point Likert-style scale. Total scores were calculated for use in improvement analyses. Participants engaging in neurofeedback for ADHD completed the ASRS [52] if they were 18 years of age or older; otherwise, they completed the ADHD-RS-IV. The GHQ-12, GAD-7, and PHQ-9 were completed by participants of all ages [55-58].

#### Continuous Performance Task

The assessment contained an 8-minute CPT, a behavioral test of response inhibition, in which participants are instructed to tap the screen when the target object (ie, an arrow-like shape pointing upward) is shown, but not when other stimuli appear. The interstimulus interval and presence of audiovisual distracter stimuli were varied throughout the task. Outcomes included average response time (RT) and response time variability (ie, the SD of RT [SDRT]), as well as omission and commission errors related to inattention and impulsivity, respectively [59]. This type of test is commonly used as an objective measure of attention and executive function [60-62] and has become a standard assessment tool for attentional difficulties [59,63,64].

#### Resting EEG

Resting EEG was recorded from 9 electrodes (ie, AF7, AF8, TP9, TP10, central [C] zero [z], frontal [F] z, F3, F4, and occipital [O] 1). The EEG assessment was divided into five sequential (ie, “sensing”) phases; in each phase, the auxiliary electrode was placed at a different scalp location: central (Cz), frontal (Fz), left (F3), right (F4), and back or posterior (O1). Each phase was split into eyes-closed and eyes-open blocks. A block continued until 30 seconds of clean EEG—sampled at 256 Hz—had been recorded, which typically took up to 45 seconds.

### Statistical Analysis

#### Symptom Questionnaires

Questionnaire results were analyzed in terms of improvement in total score from pre- to postintervention, including mean change in points, effect size (ie, Cohen *d*), and percent of users with clinically significant improvement, defined as 20% improvement [65,66]. Results are presented separately for participants scoring in healthy and abnormal ranges at baseline, as per conventional clinical cutoff values. The percent of participants who shifted from abnormal to normal (ie, healthy) ranges after the intervention is also reported. Paired-samples *t* tests (2-tailed) evaluated statistically significant improvement for each clinical measure (*P*<.05). By convention, small, medium, and large effects correspond to *d*=0.2, *d*=0.5, and *d*=0.8, respectively. For symptom questionnaires, CPT, and resting EEG analyses, multiple comparisons were corrected using the Benjamini-Hochberg (BH) method [67] to maintain a family-wise error at *P*=.05, reported as BH-adjusted *P* values (*P*_BH_). The Levene test assessed assumptions of equality of variance and corrected for inhomogeneities.

#### Continuous Performance Task

CPT results were analyzed for participants who completed child (ie, ADHD-RS-IV) or adult (ie, ASRS) ADHD questionnaires. Results are given in terms of improvement in RT and SDRT for correct responses (ie, shorter and less variable response times, respectively), commission errors, and omission errors. This includes mean change, in milliseconds or errors, and effect size. RT and SDRT scores were standardized by age to minimize age effects on performance [68]. Percent of participants demonstrating clinically significant improvement was reported, defined by a reliable change index (RCI) [69] that accounts for practice effects [70]. Exceeding a critical value of 95% for a 1-tailed test—equivalent to 1.65 SD units on a standardized *z* scale—indicates a significant reliable change, similar to others [71].

#### Resting EEG

Participants who completed the adult ADHD questionnaire at baseline were split into groups with “healthy” and “abnormal” ranges of values based on their score. Only participants with clean EEG signals were included (see Multimedia Appendix 2 for EEG preprocessing). Results were reported in terms of EEG amplitude (ie, Hz; relative power) for TAR, DAR, and TBR at baseline. Independent-samples *t* tests were conducted for each power ratio across groups (ie, healthy and abnormal values). Frequency bands were defined as follows: delta (1-4 Hz), theta (4-8 Hz), alpha (8-13 Hz), and beta (13-30 Hz). These were averaged across frontal electrodes (ie, F3 and F4, based on the frontal nodes of the frontoparietal network [25,72] and the prevalence of a clean EEG signal) during the eyes-closed condition. Improvement analyses were conducted separately for each group and included the mean change in ratio amplitude from pre- to postintervention and associated effect size; paired-samples *t* tests were used to evaluate within-group changes.

## Results

### Sample Characteristics

Data from 560 participants met the criteria for inclusion in the analysis. Depending on clinical considerations determined by their therapist, subsets of participants completed each symptom questionnaire, CPT, resting EEG, or any combination of the three. Table 1 gives sample characteristics for each assessment component, including the NFT protocols completed by 50% or more of each sample population (Multimedia Appendix 1).

**Table 1:** Sample characteristics as separated by each outcome measure and analysis.

Measure	Age (years)			Gender, n (%)			Test setting, n (%)			NFT^a^ protocols used in ≥50% of sample		No. of sessions, mean (SD)		Treatment duration (days), mean (SD)		Frequency (sessions/wk), mean (SD)	
	Mean (SD)	Range	Female		Male	Clinic		Home									
Symptom questionnaire pre-post (n=301^b^)	38 (14.5)	13-71	157 (52.7)		141 (47.3)	220 (73.1)		81 (26.9)	Reduce theta; reduce high beta; enhance low beta		53 (38.2)		91 (41.0)				4 (2.6)
CPT^c^ pre-post (ADHD^d^; n=203^e^)	37 (12.9)	13-69	103 (51.2)		98 (48.8)	112 (55.2)		91 (44.8)	Reduce theta; reduce high beta		53 (29.5)		96 (42.2)				4 (1.9)
Resting EEG^f^ baseline (adult ADHD; n=271^g^)	38 (10.9)	18-70	94 (35.2)		173 (64.8)	87 (32.1)		184 (67.9)	N/A^h^		N/A		N/A		N/A		
Resting EEG pre-post (adult ADHD; n=41^i^)	36 (9.3)	19-55	17 (42.5)		23 (57.5)	5 (12.2)		36 (87.8)	Reduce theta; enhance alpha; reduce high beta		55 (30.8)		76 (27.0)				5 (2.2)

^a^NFT: neurofeedback training.

^b^No gender identity was reported by 3 participants (n=298).

^c^CPT: continuous performance task.

^d^ADHD: attention-deficit/hyperactivity disorder.

^e^No gender identity was reported by 2 participants (n=201).

^f^EEG: electroencephalography.

^g^No gender identity was reported by 4 participants (n=267).

^h^N/A: not applicable; intervention details were not reported, as only preintervention values were of interest for baseline analyses.

^i^No gender identity was reported by 1 participant (n=40).

### Symptom Questionnaires

Results for participants who completed symptom questionnaires (n*=*301) were separated into groups with abnormal and healthy scores (Table 2). Most participants engaged in NFT protocols to reduce theta (227/301, 75.4%) and enhance high beta (248/301, 82.4%), while many who completed the PHQ-9 (76/134, 56.7%) and the ASRS (59/112, 52.7%) also performed enhanced alpha, whereas children who completed the ADHD-RS-IV also often included enhanced low beta (21/27, 78%) and enhanced sensorimotor rhythm (SMR; 16/27, 59%). In the groups with abnormal results, all questionnaires had large effect sizes (*d*=0.99 to 2.41), while the effect sizes for groups with healthy results were large only for child and adult ADHD questionnaires. Improvement in the groups with abnormal results was statistically significant for all questionnaires, with the majority (30/56, 54% to 7/7, 100%) of users demonstrating clinically significant change (ie, ≥20%) [65,66]. The most prominent improvement was observed in participants with abnormal baseline anxiety or child ADHD scores. Nevertheless, ADHD-RS-IV findings are considered preliminary given the small sample size. Most participants (30/56, 54% to 7/7, 100%) in the groups with abnormal results shifted their values to healthy ranges at postintervention. Improvement of healthy participants was statistically significant for all questionnaires, with the majority (30/66, 45% to 14/20, 70%) demonstrating clinically significant change.

**Table 2:** Improvement in self-reported subjective symptoms after ≥30 days of Myndlift neurofeedback for users that scored in the healthy range, and separately for those that scored in the abnormal range (per conventional clinical cutoffs) at baseline.

Questionnaire and group at baseline (cutoff value)			No. of sessions, mean (SD)		Treatment duration (days), mean (SD)		Change (points decreased), mean (SD)		Change *T* value		Change *P* value^a^		Effect size, *d*		Users improved by ≥20%, n (%)		Abnormal to healthy results, n (%)
**12-item General Health Questionnaire (maximum score = 36)**																	
	Abnormal (≥12; n=197)	53 (39.1)		94 (42.2)		7.8 (7.80)		13.94		<.001		0.99		139 (71)		113 (57)	
	Healthy (<12; n=66)	52 (34.3)		84 (36.6)		1.0 (4.28)		1.90		.06		0.23		30 (45)		N/A^b^	
**ADHD^c^ Rating Scale IV (for children; maximum score = 54): preliminary**																	
	Abnormal (>36; n=7)	49 (19.7)		75 (32.8)		19.3 (7.99)		6.38		<.001		2.41		7 (100)		7 (100)	
	Healthy (≤36; n=20)	53 (23.2)		102 (37.9)		7.9 (8.10)		4.36		<.001		0.98		14 (70)		N/A	
**Adult ADHD Self-Report Scale (maximum score =24)**																	
	Abnormal (≥14; n=56)	48 (25.6)		86 (41.5)		4.0 (3.81)		7.83		<.001		1.05		30 (54)		30 (54)	
	Healthy (<14; n=56)	63 (35.7)		97 (37.2)		2.1 (2.14)		7.38		<.001		0.99		33 (59)		N/A	
**7-item Generalized Anxiety Disorder scale (maximum score = 21)**																	
	Abnormal (≥14; n=99)	52 (36.7)		87 (40.2)		6.4 (5.18)		12.39		<.001		1.24		82 (83)		68 (69)	
	Healthy (<14; n=107)	55 (32.5)		97 (40.4)		1.3 (3.92)		3.43		.001		0.33		63 (59)		N/A	
**9-item Patient Health Questionnaire (max** **imum score =** **27)**																	
	Abnormal (≥10; n=63)	57 (47.6)		88 (37.7)		6.2 (5.47)		8.94		<.001		1.13		45 (71)		38 (60)	
	Healthy (<10; n=71)	57 (34.7)		95 (39.8)		1.5 (4.07)		3.04		.004		0.36		49 (69)		N/A	

^a^Reported as Benjamini-Hochberg–adjusted *P* values.

^b^N/A: not applicable; healthy subjects are already within the healthy range.

^c^ADHD: attention-deficit/hyperactivity disorder.

### Continuous Performance Task

Participants completing CPT and ADHD questionnaires performed primarily reduced theta (76/99, 77%) and enhanced high beta (81/99, 90%) protocols. Most adults also performed enhanced alpha (54/90, 60%), whereas most children also performed enhanced low beta (9/9, 100%) and enhanced SMR (7/9, 78%). Results (n*=*99) for average RT, SDRT, omission errors, and commission errors were divided by abnormal versus healthy scores for child and adult ADHD combined (Table 3). The greatest improvement observed, irrespective of group (ie, abnormal and healthy ADHD ranges), was in SDRT (*d*=1.02 and *d*=1.24, respectively), where nearly half of the participants (42/99, 43%) demonstrated clinically significant improvement, as indicated by the RCI. Although average RTs improved comparably (42/99, 43%), differences between pre- and postintervention were significant only for the healthy results group (*d*=0.56). At least one-third of users improved in their commission errors (35/99, 35%) and omission errors (45/99, 45%) from pre- to postintervention. Results from a group *(*n=104) with unknown ADHD assignment were comparable to those of groups with abnormal and healthy results (Multimedia Appendix 3).

**Table 3:** Improvement in CPT after ≥30 days of Myndlift neurofeedback (n=99) separately for healthy users that scored in the normal range for children or adults at baseline and for those in the abnormal ADHD range (per conventional clinical cutoffs).

CPT^a^ outcome and group results at baseline ASRS^b^ or ADHD^c^-RS-IV^d^		No. of sessions, mean (SD)	Treatment duration (days), mean (SD)	Change reduction, mean (SD)^e^	Change *T* value	Change *P* value^f^	Effect size, *d*	Users improved (RCI^g^ ≥1.65 SD), n (%)
**Average response time**								
	Abnormal (n=46)	48 (26.3)	85 (40.3)	8.9 (33.52)	1.80	.08	0.27	20 (43)
	Healthy (n=53)	61 (31.0)	100 (40.0)	15.0 (26.96)	4.05	<.001	0.56	22 (42)
**Response time variability (SD of response time)**								
	Abnormal (n=46)	48 (26.3)	85 (40.3)	10.3 (10.02)	6.95	<.001	1.02	18 (39)
	Healthy (n=53)	61 (31.0)	100 (40.0)	10.7 (8.64)	8.99	<.001	1.24	25 (47)
**Commission errors (impulsivity)**								
	Abnormal (n=46)	48 (26.3)	85 (40.3)	4.0 (7.23)	3.75	<.001	0.55	19 (41)
	Healthy (n=53)	61 (31.0)	100 (40.0)	2.0 (3.17)	4.51	<.001	0.62	16 (30)
**Omission errors (inattention)**								
	Abnormal (n=46)	48 (26.3)	85 (40.3)	1.5 (3.16)	3.27	.003	0.48	24 (52)
	Healthy (n=53)	61 (31.0)	100 (40.0)	0.64 (1.88)	2.48	.02	0.34	21 (40)

^a^CPT: continuous performance task.

^b^ASRS: Adult ADHD Self-Report Scale.

^c^ADHD: attention-deficit/hyperactivity disorder.

^d^ADHD-RS-IV: ADHD Rating Scale IV.

^e^Reported in milliseconds for response time average and variability, and in number of errors for commission and omission errors.

^f^Reported as Benjamini-Hochberg–adjusted *P* values.

^g^RCI: reliable change index.

### EEG Indicators of Adult ADHD

#### Resting EEG Baseline

The DAR, TAR, and TBR were calculated from baseline resting EEG data (n*=*271) in frontal regions (ie, average of F3 and F4) with eyes closed from participants scoring in abnormal (n*=*125) or healthy ranges (n=146) on the adult ADHD questionnaire.

Regarding the DAR, an independent-samples *t* test demonstrated that participants in the abnormal results group (mean 1.10, SD 0.61) had significantly greater frontal DAR than healthy participants (mean 0.90, SD 0.48; t_235_=3.02, *P*_BH_=.009, *d*=0.37). The Levene test indicated unequal variances (*F*=5.25, *P*=.02), so degrees of freedom were adjusted from 269 to 235. Post hoc independent-samples *t* tests confirmed that results were driven by participants in the abnormal results group having significantly greater frontal delta power (t_269_=2.80, *P*_BH_=.01, *d*=0.34) and less frontal alpha power (t_269_=2.61, *P*_BH_=.01, *d*=0.34) than healthy participants.

Regarding the TAR, a comparable *t* test reported a significant difference for the frontal TAR (t_269_=2.46, *P*_BH_=.02, *d*=0.30), as participants with abnormal scores (mean 0.64, SD 0.30) had significantly greater ratios than those with healthy scores (mean 0.56, SD 0.26). Post hoc *t* tests confirmed that results were driven by less frontal alpha, as opposed to differences in theta (t_269_=1.11, *P*_BH_=.27, *d*=0.13).

Regarding the TBR, a final *t* test reported no significant difference between participants with abnormal scores (mean 0.66, SD 0.27) and those with healthy scores (mean 0.64, SD 0.31; t_269_=0.532, *P*_BH_=.60, *d*=0.06).

#### Preliminary Resting EEG Improvement

Changes in the DAR, TAR, and TBR in the frontal regions with eyes closed were reported for participants (n=41) scoring in the abnormal (n=20) or healthy ranges (n=21) of the adult ADHD questionnaire (Table 4). Most participants completed reduced theta (32/41, 78%), enhanced high beta (37/41, 90%), and enhanced alpha protocols (27/41, 66%). After correcting for multiple comparisons, significant improvement was only reported for the DAR in the abnormal results group.

**Table 4:** Change in resting EEG ratios from frontal (ie, average F3 and F4) electrodes during the eyes-closed condition after ≥30 days of Myndlift neurofeedback (n=41) for healthy users and separately for those that scored in the abnormal adult ADHD range (per conventional clinical cutoffs) at baseline.

EEG^a^ pre-post outcome and group at baseline (cutoff value)			No. of sessions, mean (SD)		Treatment duration (days), mean (SD)		Change reduction (Hz), mean (SD)		Change *T* value		Change *P* value^b^		Effect size, *d*
**Delta/alpha ratio**													
	Abnormal (≥14; n=20)	49 (22.1)		77 (26.8)		0.20 (0.284)		3.15		.03		0.70	
	Healthy (<14; n=21)	61 (36.9)		76 (27.8)		0.08 (0.450)		0.79		.59		0.18	
**Theta/alpha ratio**													
	Abnormal (≥14; n=20)	49 (22.1)		77 (26.8)		0.04 (0.171)		1.00		.66		0.22	
	Healthy (<14; n=21)	61 (36.9)		76 (27.8)		0.01 (0.227)		0.35		.66		0.08	
**Theta/beta ratio**													
	Abnormal (≥14; n=20)	49 (22.1)		77 (26.8)		0.04 (0.144)		1.34		.79		0.30	
	Healthy (<14; n=21)	61 (36.9)		76 (27.8)		0.02 (0.218)		0.44		.73		0.10	

^a^EEG: electroencephalography.

^b^Reported as Benjamini-Hochberg–adjusted *P* values.

## Discussion

### Principal Findings

This retrospective study offers initial evidence of therapist-guided remote neurofeedback as an effective tool for reducing subjective symptoms, improving objective cognitive performance, and adaptively modifying EEG markers. Improvements in attention were evident in children and adults with ADHD, as well as healthy participants. Findings suggest that the TBR is not a reliable marker for adult ADHD, instead demonstrating alternative elevated slow/fast power ratios [37]. Moreover, we provide preliminary evidence for improvement (ie, reduced DAR) in adults with ADHD. These findings offer a promising use for remote NFT as a low-cost alternative to clinic-based EEG.

### Efficacy for Improving Mental Health Remotely

Based on real-world data, significant improvement was reported across standardized questionnaires. The greatest improvement was observed in participants with abnormal anxiety scores, where most received reduced theta, enhanced high beta, and enhanced alpha protocols. As anticipated, greater effect sizes were observed for participants with scores in the abnormal versus healthy ranges. Interestingly, healthy participants and those with ADHD, both children and adults, demonstrated significant improvement with large effect sizes after completing primarily reduced theta and enhanced high beta protocols, as well as adults who completed enhanced alpha protocols or children who completed reduced low beta and reduced SMR protocols. Consistent with the literature [33-35], our findings suggest that children and adults may benefit from unique NFT protocols to improve ADHD symptoms, although a larger sample is required to confirm preliminary ADHD-RS-IV results.

Apart from the child ADHD assessment, questionnaire analyses included large total sample numbers (ie, 112 to 263 participants), and after an average of 53 NFT sessions, 57% to 78% of the participants demonstrated significant improvement, depending on the questionnaire. Results were particularly impressive compared to other in-app mental health therapeutics [73-76], such as mobile-enabled text psychotherapy [77] or app-based cognitive behavioral therapy [78]. The majority (61%) of participants scoring in the abnormal ranges moved to the healthy results group over an average of approximately 3 months, a time frame costing less than US $1500 with Myndlift versus US $6000 to $8000 for traditional neurofeedback.

### Improved Cognitive Performance for Healthy Participants and Those With ADHD

NFT led to greater consistency in response times on a response inhibition task for subjects scoring in healthy or abnormal ADHD ranges, agreeing with similar reports of subjects with ADHD [79,80]. In addition, the RCI demonstrated that approximately 50% of healthy participants improved their average response time, while similarly, participants in abnormal ranges reduced omission errors. Importantly, CPT findings agree with improved ADHD questionnaire scores, suggesting that NFT provides objective evidence of improved executive function, the primary cognitive domain impacted by attentional difficulties.

### Identifying Adult ADHD Neuromarkers

Resting EEG findings demonstrated that elevated DAR and TAR were indicative of adult ADHD at baseline. This translated to significantly higher levels of delta and lower levels of alpha, as previously reported in adults with ADHD [39-42,81,82]. Notably, Liechti and colleagues [35] reported high theta to be less consistent in adults than in children, and that ADHD versus healthy control classification improved having exploratorily included delta waves in the discriminant analysis. Adults with ADHD may present slower theta waves—bordering fast delta waves—than children, although further analysis is required. Together, findings are consistent with the cortical hypoarousal theory, where low-power fast oscillations accompany reduced self-control and executive functioning [83], and high-power slow oscillations are reported with decreased subcortical motivational drive [84]. Preliminary evidence for reduced DAR in adult ADHD from pre- to postassessment may reflect the improved ADHD symptoms and CPT measures, particularly given the success of protocols inhibiting slow oscillations and enhancing fast oscillation [1], and the high percentage of ADHD participants performing reduced theta (ie, slow) and enhanced alpha (ie, fast) protocols.

In contrast to our work and that of others, several groups reported high alpha power at baseline during eyes-closed conditions in adult ADHD populations [85,86], or rather, no difference across ADHD participants and healthy controls [87,88]. Importantly, variability across the adult ADHD literature may, in part, be due to the heterogeneity of ADHD [34,35] and differences in study designs, sample sizes, analyses, and EEG technology [89]. For example, Loo and colleagues [38] demonstrated that adults with ADHD combined-type (ie, symptoms of inattention and hyperactivity or impulsivity) present reduced alpha power globally, compared to ADHD inattentive-type or non-ADHD controls.

### Limitations and Future Directions

Study results are encouraging, but conclusions should be tempered by limitations, including small subgroup sample sizes and lack of control groups. Moreover, subjects may have received alternative treatment in parallel (eg, medication) that could influence symptom improvement as well as alter neuromarkers. For example, two studies administering stimulants (ie, methylphenidate or dexamphetamine) to treat symptoms of ADHD in adults demonstrated altered delta [90,91] and theta waves [90] posttreatment. No changes in alpha or beta waves were reported. Given the evidence in this study for altered delta and alpha waves in adults with abnormal ADHD scores, we would hypothesize that the mechanism of action for stimulants versus NFT may differ*, resulting in influence over varied frequency bands*. Moreover, as this population reflects real-world use, the likelihood of these two forms of treatment to have commenced simultaneously, for treating symptoms of depression, anxiety, and ADHD, would arguably be low. Those seeking treatment with remote neurofeedback most often do so to avoid taking pharmaceuticals [92,93] or, rather, to supplement their current treatment, which alone may not be sufficiently effective [94]. Frank H Duffy [95], a Harvard professor and pediatric neurologist, suggests that “if any medication had demonstrated such a wide spectrum of efficacy it would be universally accepted and widely used.” Further, controlled research studies will be required to facilitate comparison of neurofeedback efficacy with other interventions. Notwithstanding these limitations, the findings are essential as they reflect real-world benefits of remote neurofeedback to actual patients. Follow-up analyses will compare benefits across NFT protocols and will further evaluate the impact on resting EEG outcomes.

### Conclusions

Preliminary findings from this retrospective pilot study demonstrate efficacy of remote NFT in improving mental health, particularly for individuals with symptoms of ADHD and anxiety, mainly through reduced theta, enhanced high beta, and enhanced alpha NFT protocols. Moreover, adult ADHD was distinguished from healthy individuals by elevated frontal DARs, where ratios were significantly reduced following NFT. The effectiveness of the system in a real-world population via remote use positions it as an affordable and accessible alternative to clinic-based systems.

## Appendix

Multimedia Appendix 1 Neurofeedback protocols implemented in this study.

Multimedia Appendix 2 Electroencephalography preprocessing.

Multimedia Appendix 3 Continuous performance task improvement for users without an ADHD assessment. ADHD: attention-deficit/hyperactivity disorder.

## Abbreviations

ADHD: attention-deficit/hyperactivity disorder
ADHD-RS-IV: ADHD Rating Scale IV
AF: anterior frontal
ASRS: Adult ADHD Self-Report Scale
BH: Benjamini-Hochberg
C: central
CPT: continuous performance task
DAR: delta/alpha ratio
DSM-5: Diagnostic and Statistical Manual of Mental Disorders, Fifth Edition
EEG: electroencephalography
F: frontal
GAD-7: 7-item Generalized Anxiety Disorder scale
GHQ-12: 12-item General Health Questionnaire
IRB: Institutional Review Board
NFT: neurofeedback training
O: occipital
*P*_BH_: Benjamini-Hochberg–adjusted *P* value
PHQ-9: 9-item Patient Health Questionnaire
RCI: reliable change index
RT: response time
SDRT: SD of response time, response time variability
SMR: sensorimotor rhythm
TAR: theta/alpha ratio
TBR: theta/beta ratio
TP: temporal pole
z: zero
