# Supplementary material for: Preliminary Real-World Evidence Supporting the Efficacy of a Remote Neurofeedback System in Improving Mental Health: Retrospective Single-Group Pretest-Posttest Study
Source: JMIR Form Res. 2022 Jul 8;6(7):e35636. doi: 10.2196/35636 (PMC9308076; doi:10.2196/35636)
Supplement: Multimedia Appendix 1 [file formative_v6i7e35636_app1.docx]

**Multimedia Appendix 1**

**Neurofeedback Protocols Implemented**

Customized neurofeedback protocols were created by participants’ clinicians, based on their relevant needs. Supplementary Table 1 provides a summary of protocols completed by participants. Notably, most participants completed several protocols, most often, reducing theta and high beta power. Additionally, protocols for participants who completed the PHQ-9 (depression questionnaire) and/or adults who performed ADHD related assessments (ASRS questionnaire, pre-post CPT and resting EEG) often also included alpha power enhancement. Instead, children with ADHD often also performed protocols with low beta and/or sensorimotor rhythm (SMR) enhancement. Collectively, the remaining protocols were completed by less than a third of participants in addition to the main protocols used. These included enhanced or inhibited delta, enhanced theta, inhibited alpha, inhibited low alpha, and enhanced high beta, inhibited SMR, inhibited high beta, enhanced, or inhibited gamma. Frequency bands are defined as follows: delta (1-4 Hz), theta (4-8 Hz), low alpha (8-10 Hz), high alpha (10-13 Hz), alpha (8-13 Hz), low beta (13-21 Hz), high-beta (21-30 Hz), beta (13-30Hz) and gamma (30-45 Hz).

**Table S1.** Percentage of Myndlift users who completed each NFT protocol, as separated by analysis and participant subgroup (abnormal or healthy group, determined at baseline, or unknown assignment). Arrows indicate enhancement (**↑**) or inhibition (**↓**) for each power band, while red text denotes NFT protocols that were completed by ≥50% of participants (in a given subgroup).

| **Pre-post Assessment** | **Group at Baseline** | ***N*** | **NFT Protocols Used (% of Subgroup Population)** | | | | | |
| --- | --- | --- | --- | --- | --- | --- | --- | --- |
|  |  |  | **↓ Theta** | **↑ Alpha** | **↑ SMR** | **↑ Low Beta** | **↓ High Beta** | **Other*** |
| General Health Questionnaire (GHQ-12; max. 36) | Abnormal | 197 | 76% | 45% | 42% | 47% | 82% | 28% |
|  | Healthy | 66 | 76% | 42% | 47% | 50% | 79% | 24% |
| ADHD Rating Scale for Children (ADHD-RS-IV; max. 54) | Abnormal | 7 | 100% | 14% | 43% | 86% | 71% | 29% |
|  | Healthy | 20 | 100% | 25% | 65% | 75% | 80% | 5% |
| ADHD Rating Scale for Adults (ASRS; max. 24) | Abnormal | 56 | 73% | 50% | 41% | 45% | 86% | 30% |
|  | Healthy | 56 | 80% | 55% | 36% | 38% | 89% | 27% |
| Anxiety Scale (GAD-7; max. 21) | Abnormal | 99 | 76% | 53% | 31% | 38% | 88% | 24% |
|  | Healthy | 107 | 74% | 46% | 39% | 46% | 85% | 32% |
| Depression Scale (PHQ-9; max. 27) | Abnormal | 63 | 78% | 52% | 32% | 37% | 89% | 32% |
|  | Healthy | 71 | 75% | 61% | 31% | 35% | 92% | 28% |
| CPT - Adult ADHD | Abnormal | 44 | 68% | 57% | 36% | 39% | 86% | 30% |
|  | Healthy | 46 | 80% | 63% | 30% | 33% | 93% | 24% |
|  | Unknown | 95 | 62% | 45% | 40% | 47% | 71% | 33% |
| CPT - Child ADHD | Abnormal | 2 | 100% | 0% | 100% | 100% | 100% | 0% |
|  | Healthy | 7 | 100% | 0% | 71% | 100% | 86% | 0% |
|  | Unknown | 9 | 67% | 22% | 56% | 67% | 67% | 22% |
| EEG - Adult ADHD | Abnormal | 20 | 80% | 75% | 20% | 20% | 95% | 25% |
|  | Healthy | 21 | 76% | 57% | 29% | 33% | 86% | 33% |

**Other* includes enhance or inhibit delta, enhance theta, inhibit alpha, inhibit low alpha and enhance high beta, inhibit SMR, inhibit high beta, enhance or inhibit gamma
