# Supplementary material for: Preliminary Real-World Evidence Supporting the Efficacy of a Remote Neurofeedback System in Improving Mental Health: Retrospective Single-Group Pretest-Posttest Study
Source: JMIR Form Res. 2022 Jul 8;6(7):e35636. doi: 10.2196/35636 (PMC9308076; doi:10.2196/35636)
Supplement: Multimedia Appendix 2 [file formative_v6i7e35636_app2.docx]

**Multimedia Appendix 2**

**EEG Preprocessing**

Data were collected from participants across several continents, where offline preprocessing included (in the following order) FIR filtering (4-40Hz), the appropriate 50-Hz (Europe and Asia) or 60-Hz (North America) notch filters depending on recording location, re-referencing to Tp10 electrode. Artifacts were detected by dividing the Continuous EEG into 1-second epochs (256 samples). A custom algorithm determined whether epochs were valid for inclusion based on signal characteristics (power, variance, amplitude etc.) of the epoch. Recordings with ≥40% identified artifacts were rejected, leaving 66.4% of the total recordings (88,029) valid. Only recordings from relevant users (that met inclusion criteria and presented a clean EEG signal) were used in analyses. For each recording, Python’s scipy package fft function was used to compute a power spectrum with a frequency resolution of 1 Hz. Total power (µV2) was calculated for the delta (1–3 Hz), theta (4–7 Hz), alpha (8–12 Hz), beta (13–30 Hz) bands. Band power was then normalized by the total power of the signal.
