# Supplementary material for: Preliminary Real-World Evidence Supporting the Efficacy of a Remote Neurofeedback System in Improving Mental Health: Retrospective Single-Group Pretest-Posttest Study
Source: JMIR Form Res. 2022 Jul 8;6(7):e35636. doi: 10.2196/35636 (PMC9308076; doi:10.2196/35636)
Supplement: Multimedia Appendix 3 [file formative_v6i7e35636_app3.docx]

**Multimedia Appendix 3**

**Table S1.** Improvement in continuous performance task (CPT) after ≥30 days of Myndlift neurofeedback (*n*=104) separately for users that did not complete an ADHD questionnaire.

| **CPT Outcome** | **Group at Baseline**  **(cut-off value)**  **ASRS or ADHD-RS-IV** | ***n*** | **Mean ± SD Number of Sessions** | **Mean ± SD Treatment duration (days)** | **Mean ± SD** **change ↓** | **Change *T*-value** | ***Change p*-value (*p_BH_)*** | **Effect size (*d*)** | **Percent users improved (RCI ≥ 1.65 SD)** |
| --- | --- | --- | --- | --- | --- | --- | --- | --- | --- |
| Average Response Time (RT) | Unknown | 104 | 52 ± 29.5 | 100 ± 43.6 | 20.5 ± 25.90 ms | 8.06 | <.001 | .79 | 48% |
| Response Time Variability (SDRT) | Unknown | 104 | 52 ± 29.5 | 100 ± 43.6 | 9.6 ± 13.39 ms | 7.33 | <.001 | .72 | 47% |
| Commission errors (impulsivity) | Unknown | 104 | 52 ± 29.5 | 100 ± 43.6 | 2.0 ± 6.53 err. | 3.08 | .004 | .30 | 28% |
| Omission errors (inattention) | Unknown | 104 | 52 ± 29.5 | 100 ± 43.6 | .37 ± 2.26 err. | 1.70 | .09 | .17 | 34% |

RCI: Reliable change index
